# The Black Death in the Fourteenth Century

**Published:** 1833-07-01

**Authors:** 


### VIII.

The Black Death in the Fourteenth Century. From the German of *J. F. C. Hecker*, M.D. &c. Translated by *B. G. Babington*, M.D. 12mo. pp. 205, Schloss, 1833.

The translator observes that one and an important motive for laying this narrative before the English public is, that by comparing the sufferings of past ages with those of our own time, we may be made sensible how lightly the chastening hand of Providence has fallen on the present generation, and how much reason, therefore, we have to feel grateful for the mercy shewn us. This is a worthy and a Christian motive, and will, we have no doubt, be properly appreciated. We are induced to notice the little work for a reason more mundane, more tainted with human interests and human learning; to shew how, under Providence, civilization protects man from those terrific visitations of disease, which, in earlier ages, were characterized alike by ferocity and frequency, and whose returns were looked for with almost as much regularity as that of the seasons. When we read of a pestilence which swept away one quarter of the population of the old world, in the short space of four years, we read of that which reason tells, or seems to tell, us will not again occur, unless the Almighty inflict it as a special evidence of his wrath, or the circumstances of the world lapse back into the foul and unwholesome condition of the middle ages. We confess, then, that we do not entertain the alarm which some experience or affect to shew at the prospect of coming or of future plagues. Restore London to the condition in which it was antecedently to the great fire, or to the condition in which Europe was at the time of the black death, and then, indeed, we will join them in their bodings, and participate in their anxiety. But, until that is done, we must persist in thinking of approaching plagues and approaching dangers as we thought of the approaching cholera, that if they do visit this happy isle, they will come shorn of their most terrific features, and wither before the influence of cleanliness, comfort, a wholesome diet, and a salubrious atmosphere.

Many of our readers may be curious to learn what the black death of the fourteenth century was. The fictitious horrors of Defoe melt into nothingness when compared with its realities, and the reader of the narrative of its destructive progress, feels compelled to suspect that much exaggeration must be mixed with the details. We shall endeavour to give a short but connected account of this fearful scourge. Not pursuing the order which Dr. Hecker and his translator have adopted, we shall first describe its origin and progress.

It first appeared in China; but its exact nature is unknown. From China it passed to the western countries of Asia, where it shewed itself as the Oriental plague, with inflammation of the lungs. It was thought to be carried to Constantinople, then the seat of the Eastern Empire, and the centre of commerce between the East and West, from the Northern coast of the Black Sea, after it had depopulated the countries between those routes of commerce.

"It appeared as early as 1347, in Cyprus, Sicily, Marseilles and some of the seaports of Italy. The remaining islands of the Mediterranean, particularly Sardinia, Corsica and Majorca, were visited in succession. Foci of contagion existed also in full activity along the whole southern coast of Europe; when, in January, 1348, the plague appeared in Avignon,* and in other cities in the south of France and north of Italy, as well as in Spain.

The precise days of its eruption in the individual towns, are no longer to be ascertained; but it was not simultaneous: for in Florence, the disease appeared in the beginning of April;† in Cesena, the 1st of June;‡ and place after place was attacked throughout the whole year; so that the plague, after it had passed through the whole of France and Germany, where, however, it did not make its ravages until the following year, did not break out till August, in England; where it advanced so gradually, that a period of three months elapsed before it reached London.|| The Northern Kingdoms were attacked by it in 1349. Sweden, indeed, not until November of that year: almost two years after its eruption in Avignon.§ Poland received the plague in 1349, probably from Germany,¶ if not from the northern countries; but in Russia, it did not make its appearance until 1351, more than three years after it had broken out in Constantinople. Instead of advancing in a north-westerly direction from Tauris and from the Caspian Sea, it had thus made the great circuit of the Black Sea, by way of Constantinople, Southern and Central Europe, England, the Northern Kingdoms and Poland, before it reached the Russian territories; a phenomenon which has not again occurred with respect to more recent pestilences originating in Asia." 52.

It would seem that unusual phenomena, violent convulsions of the earth, and unnatural and unwholesome conditions of the atmosphere, were observed before the outbreak and during the continuance of this black death. We have little doubt that many of these reputed facts are but idle tales and bold impostures, the offspring of knavery, terror, or credulity. Yet, making every allowance for the operation of these causes, it must be owned that there is still a mass of accredited and probable facts, which go to prove that some great atmospheric or terrestrial agencies were at work, contaminating all nature, and fraught with destruction to man.

"The series of these great events began in the year 1333, fifteen years before the plague broke out in Europe: they first appeared in China. Here a parching drought, accompanied by famine, commenced in a tract of country watered by the rivers Kiang and Hoai. This was followed by such violent torrents of rain, in and about Kingsai, at that time the capital of the Empire, that, according to tradition, more than 400,000 people perished in the floods. Finally, the mountain Tsincheo fell in, and vast clefts were formed in the earth. In the succeeding year (1334), passing over fabulous traditions, the neighbourhood of Canton was visited by inundations; whilst in Tche, after an unexampled drought, a plague arose, which is said to have carried off about 5,000,000 of people. A few months afterwards an earthquake followed, at and near Kingsai; and subsequent to the falling in of the mountains of Ki-ming-chan, a lake was formed of more than a hundred leagues in circumference, where, again, thousands found their grave. In Hou-kouang and Ho-han, a drought prevailed for five months; and innumerable

---

* *Guid. Cauliac,* Loc. cit.

† *Matt. Villani,* Istorie, in *Muratori,* T. XIV. p. 14.

‡ Annal. Caesenat, *Ibid.* p. 1179.

|| *Barnes.* Loc. cit.

§ *Olof Dalin's,* Svea-Rikes Historie, III. vol. *Stockholm,* 1747—61, 4. Vol. II. C. 12, p. 496.

¶ *Dlugoss,* Histor. Polon. L. IX. p. 1086, T. I. *Lips.* 1711, fol.

swarms of locusts destroyed the vegetation; while famine and pestilence, as usual, followed in their train. Connected accounts of the condition of Europe before this great catastrophe, are not to be expected from the writers of the fourteenth century. It is remarkable, however, that simultaneously with a drought and renewed floods in China, in 1336, many uncommon atmospheric phenomena, and in the winter, frequent thunderstorms, were observed in the north of France; and so early as the eventful year of 1333, an eruption of Etna took place.* According to the Chinese annals, about 4,000,000 of people perished by famine in the neighbourhood of Kiang in 1337; and deluges, swarms of locusts, and an earthquake which lasted six days, caused incredible devastation. In the same year, the first swarms of locusts appeared in Franconia, which were succeeded in the following year by myriads of these insects. In 1338, Kingsai was visited by an earthquake of ten days' duration; at the same time France suffered from a failure in the harvest; and thenceforth, till the year 1342, there was in China, a constant succession of inundations, earthquakes, and famines. In the same year great floods occurred in the vicinity of the Rhine and in France, which could not be attributed to rain alone; for, everywhere, even on the tops of mountains, springs were seen to burst forth, and dry tracts were laid under water in an inexplicable manner. In the following year, the mountain Hong-tchang, in China, fell in, and caused a destructive deluge; and in Pien-tcheou and Leang-tcheou, after three months' rain, there followed unheard-of inundations, which destroyed seven cities. In Egypt and Syria, violent earthquakes took place; and in China they became, from this time, more and more frequent; for they recurred, in 1344, in Ven-tcheou, where the sea overflowed in consequence; in 1345, in Ki-tcheou, and in both the following years in Canton, with subterraneous thunder. Meanwhile, floods and famine devastated various districts, until 1347, when the fury of the elements subsided in China."†

Nature thus convulsed, it is no wonder that pestilences should arise, or that, having arisen, they should be widely spread. The signs of terrestrial commotions commenced in Europe in 1348, and the same dark drama of earthquakes, floods, failures of crops, and famine, was enacted to the terrified Europeans, which had been already played on a more costly scale to the Asiatic nations. In the island of Cyprus the plague had already broken out when a pestiferous wind spread so poisonous an odour, that many being overpowered by it fell down suddenly and expired in dreadful agonies. This *simoom* was succeeded by an earthquake, a slaughter of their slaves by the Cypriots, and a fearful hurricane. German accounts say that a thick stinking mist advanced from the East and spread itself over Italy, and then followed the earthquakes, famines, &c., to which we have already alluded, and which we need not more particularly describe than by saying that almost every European nation would seem to have had its share. Enough has been written and said to convince all unprejudiced persons that whatever was the cause or the mode of propagation of the plague, there were great atmospherical and telluric influences set in motion, which, we may fairly suppose, to be powerful in their effects on many, although we know not precisely what those effects are.

---

* V. Hoff. Geschichte der naturlichen Veranderungen der Erdoberflache, T. II. p. 264. Gotha, 1824. This eruption was not succeeded by any other in the same century, either of Etna or of Vesuvius.

† Deguignes Loc. cit. p. 226, from Chinese sources.

But what was the disease—what its symptoms—what its character? It was an oriental plague, marked by inflammatory boils and tumors of the glands, such as break out in no other febrile disease. From these boils, and from the black spots which appeared upon the skin, it received in Germany its name of Black Death, and in Italy that of the Great Mortality. The following account is given of the symptoms by the imperial writer Kantakusenos, whose own son, Andronikus, perished by it.

"Great imposthumes* of the thighs and arms of those affected, which, when opened, afforded relief by the discharge of an offensive matter. Buboes, which are the infallible signs of the oriental plague, are thus plainly indicated, for he makes separate mention of smaller boils on the arms and in the face, as also in other parts of the body, and clearly distinguishes these from the blisters,† which are no less produced by plague in all its forms. In many cases, black spots‡ broke out all over the body, either single, or united and confluent.

These symptoms were not all found in every case. In many, one alone was sufficient to cause death, while some patients recovered, contrary to expectation, though afflicted with all. Symptoms of cephalic affection were frequent; many patients became stupified and fell into a deep sleep, losing also their speech from palsy of the tongue; others remained sleepless and without rest. The fauces and tongue were black, and as if suffused with blood; no beverage would assuage their burning thirst, so that their sufferings continued without alleviation until terminated by death, which many in their despair accelerated with their own hands. Contagion was evident, for attendants caught the disease of their relations and friends, and many houses in the capital were bereft even of their last inhabitant. Thus far the ordinary circumstances only of the oriental plague occurred. Still deeper sufferings, however, were connected with this pestilence, such as have not been felt at other times; the organs of respiration were seized with a putrid inflammation; a violent pain in the chest attacked the patient; blood was expectorated, and the breath diffused a pestiferous odour." 7.

In the West the eruption of the disease was marked by an ardent fever, accompanied by an evacuation of blood, which proved fatal in the first three days. Buboes and inflammatory boils did not at first come out at all, but the disease, in the form of carbuncular affection of the lungs, destroyed before the other symptoms were developed. In Avignon it is said that even the vicinity of those who had fallen ill of plague was certain death, and, as a natural and usual consequence of contagion or a belief in it, parents abandoned their infected children, and all the ties of kindred were dissolved. Such is the demoralization of pestilence. After this period buboes in the axilla and in the groin, and inflammatory boils all over the body made their appearance; but it was not till some months afterwards that some patients recovered with matured buboes, as in the ordinary milder form of plague. Boccacio, who was an eye-witness of its fatality in Florence, gives a lively description of it. It commenced here, not as in the East, with bleeding at the nose, a sure sign of inevitable death; but there took place at the beginning, both in men and women, tumours in the groin and in the axilla, varying in circumference up to the size of an apple or an egg, and called by the people, pest-boils (gavoccioli). Then there appeared similar tumours indiscriminately over all parts of the body, and black or blue spots came out on the arms or thighs, or on other parts, either single and large, or

---

small and thickly studded. These spots proved equally fatal with the pest-boils, which had been from the first regarded as a sure sign of death. No power of medicine brought relief—almost all died within the first three days, some sooner, some later, after the appearance of these signs, and for the most part entirely without fever or other symptoms. The plague spread itself with the greater fury, as it communicated from the sick to the healthy, like fire among dry and oily fuel, and even contact with the clothes and other articles which had been used by the infected, seemed to induce the disease. As it advanced, not only men, but animals fell sick and shortly expired, if they had touched things belonging to the diseased or dead. Thus Boccacio himself saw two hogs on the rags of a person who had died of plague, after staggering about for a short time, fall down dead, as if they had taken poison. In other places, multitudes of dogs, cats, fowls and other animals, fell victims to the contagion; and it is to be presumed that other epizootes among animals likewise took place, although the ignorant writers of the fourteenth century are silent on this point.

In Germany it was not so fatal as in other parts of Europe. Many sudden deaths occurred on the coasts of the North Sea and Westphalia, without any further development of the malady. In France, which it entered by Avignon, it was more destructive than in Germany, not more than two in twenty of the inhabitants of many places surviving. In England it was very fatal, only a tenth part of the inhabitants being reported to have escaped. Here the eyes of the patients were considered as sources of contagion, which had the power of acting at a distance. We feel surprised that some of our contagionists who took such pains, on a recent occasion, to consult Defoe, did not also amalgamate this ancient notion with their creed. In Norway not more than a third of the inhabitants were spared, and ships without crews were seen here, as in the Mediterranean, drifting about and thrown on shore. The plague did not appear in Russia till two years later than in Southern Europe.

It is evident, as Dr. Hecker remarks, that this disease was in its important features identical with the oriental plague. The inflammations of the lungs that were frequently observed were probably of the character of those pleuro-pneumonies that occur in all low fevers. In considering the mortality, we should not forget the condition of European Towns at that period. They were, with few exceptions, narrowly built, kept in a filthy state, and surrounded with stagnant ditches, and it is expressly stated, with respect to Avignon and Paris, that uncleanliness of the streets increased the plague considerably. Dr. Hecker's description of the people of the 14th century is as striking as it is true. They were, says he, but little civilized. The church had indeed subdued them; but they all suffered from the ill-consequences of their original rudeness. The dominion of the law was not yet confirmed. Sovereigns had everywhere to combat powerful enemies to internal tranquillity and security. The cities were fortresses for their own defence. Marauders encamped on the roads.—The husbandman was a feodal slave, without possessions of his own.—Rudeness was general.—Humanity, as yet unknown to the people.—Witches and heretics were burned alive. Gentle rulers were contemned as weak;—wild passions, severity and cruelty, everywhere predominated.—Human life was little regarded.—Governments concerned not themselves about the numbers of their subjects, for whose welfare it was incumbent on them to provide.

It is difficult to form an accurate estimate of the mortality. Kairo lost daily, when the plague was at its height, from 10 to 15,000. In China, more than 13,000,000 are said to have perished. In Aleppo 500 died daily—22,000 people, and most of the animals, were carried off in Gaza, and it was reported to Pope Clement, at Avignon, that throughout the East, probably with the exception of China, 23,840,000 persons had fallen victims. The following is supposed to be a probable statement of the mortality, in many of the greater European cities.

| | | | |
|---|---|---|---|
| In Florence there died of the Black Plague | 60,000 | In Basle | 14,000 |
| In Venice | 100,000 | In Erfurt, at least | 16,000 |
| In Marseilles, in one month | 16,000 | In Weimar | 5,000 |
| In Siena | 70,000 | In Limburg | 2,500 |
| In Paris | 50,000 | In London, at least | 100,000 |
| In St. Denys | 14,000 | In Norwich | 51,100 |
| In Avignon | 60,000 | To which may be added— | |
| In Strasburg | 16,000 | Franciscan Friars in Germany | 124,434 |
| In Lubeck | 9,000 | Minorities in Italy | 30,000 |

Leaving all further accounts of specific mortality we may allude to a few circumstances which tend to throw light on the workings of human nature, and should convey a lesson to rulers, if rulers could ever learn. In many places it was rumoured that plague patients were buried alive. We know how similar rumours prevailed during the recent prevalence of the cholera. Morals were deteriorated every where, and the service of God was in a great measure laid aside in England. Covetousness became general, and when tranquillity was restored the great increase of *lawyers* was astonishing. In Russia the fear of contagion made fathers and mothers desert their children, and children their parents.

Two extraordinary circumstances were connected with this plague:—the spread of the doctrines and increase of the numbers of the Flagellants, and the persecution of the Jews. We have seen even in our own day, religious fanaticism derive strength from the epidemic cholera, and in Paris, as elsewhere, the Jews have been accused of poisoning the wells. The human mind is the same in all ages, and when actuated by impulses so overwhelming as panic and despair, the same dark passions and bloody resolves would appear to arise as a natural and general consequence. The processions of the Flagellants were merely political in their consequences and secret objects, and vicious in their palpable effects, but the persecution of the Jews is a circumstance so remarkable, so illustrative of the black side of human nature, a drama at once so cruel and so instructive, that we cannot forbear a shuddering glance at it.

"The persecution of the Jews commenced in September and October, 1348,* at Chillon, on the Lake of Geneva, where the first criminal proceedings were instituted against them, after they had long before been accused by the people of poisoning the wells; similar scenes followed in Bern and Freyburg, in January, 1349. Under the influence of excruciating suffering, the tortured Jews confessed

* "See the original proceedings, in the Appendix."

themselves guilty of the crime imputed to them; and it being affirmed that poison had in fact been found in a well at Zoffingen, this was deemed a sufficient proof to convince the world; and the persecution of the abhorred culprits, thus appeared justifiable. Now, though we can take as little exception at these proceedings, as at the multifarious confessions of witches, because the interrogatories of the fanatic and sanguinary tribunals were so complicated, that by means of the rack, the required answer must inevitably be obtained; and it is besides conformable to human nature, that crimes which are in every body's mouth, may, in the end, be actually committed by some, either from wantonness, revenge, or desperate exasperation: yet crimes and accusations, are, under circumstances like these, merely the offspring of a revengeful, frenzied, spirit in the people; and the accusers, according to the fundamental principles of morality, which are the same in every age, are the more guilty transgressors.

Already in the Autumn of 1348, a dreadful panic caused by the supposed poisoning, seized all nations; and in Germany especially, the springs and wells were built over, that nobody might drink of them, or employ the water for culinary purposes; and for a long time the inhabitants of numerous towns and villages, used only river and rain water.* The city gates were also guarded with the greatest caution,—only confidential persons were admitted; and if medicine, or any other article which might be supposed to be poisonous, was found in the possession of a stranger,—and it was natural that some should have these things by them for their private use,—they were forced to swallow a portion of it.† By this trying state of privation, distrust and suspicion, the hatred against the supposed poisoners became greatly increased, and often broke out in popular commotions, which only served still further to infuriate the wildest passions. The noble and the mean, fearlessly bound themselves by an oath to extirpate the Jews by fire and sword, and to snatch them from their protectors, of whom the number was so small, that throughout all Germany but few places can be mentioned where these unfortunate people were not regarded as outlaws—martyred and burnt.‡ Solemn summonses were issued from Bern to the towns of Basle, Freyburg in the Breisgau, and Strasburg, to pursue the Jews as poisoners. The Burgomasters and Senators, indeed, opposed this requisition; but in Basle the populace obliged them to bind themselves by an oath, to burn the Jews, and to forbid persons of that community from entering their city, for the space of two hundred years. Upon this, all the Jews in Basle, whose number could not have been inconsiderable, were enclosed in a wooden building, constructed for the purpose, and burnt together with it, upon the mere outcry of the people, without sentence or trial, which indeed would have availed them nothing. Soon after, the same thing took place at Freyburg. A regular diet was held at Bennefeld, in Alsace, where the bishops, lords and barons, as also deputies of the counts (*query*, counties?) and towns, consulted how they should proceed with regard to the Jews; and when the deputies of Strasburg—not indeed the bishop of this town, who proved himself a violent fanatic—spoke in favor of the persecuted, as nothing criminal was substantiated against them; a great outcry was raised, and it was vehemently asked, why, if so, they had covered their wells and removed their buckets? A sanguinary decree was resolved upon, of which the populace, who obeyed the call of the nobles and superior clergy, became but the too willing executioners.§ Wherever the Jews were not burnt, they were at

* "*Hermanni Gygantis* Flores temporum, sive Chronicon Universale—*Ed. Meuschen.* Lugdun, Bat. 1743. 4to. p. 139. Hermann, a Franciscan monk of Franconia, who wrote in the year 1349, was an eye-witness of the most revolting scenes of vengeance throughout all Germany."

† "*Guid. Cauliac.* Loc. cit."

‡ "*Hermann.* Loc. cit."

§ "*Albert Argentin—Königshoven,* Loc. cit."

least banished; and so, being compelled to wander about, they fell into the hands of the country people, who without humanity, and regardless of all laws, persecuted them with fire and sword. At Spires, the Jews, driven to despair, assembled in their own habitations, which they set on fire, and thus consumed themselves with their families. The few that remained were forced to submit to baptism; while the dead bodies of the murdered, which lay about the streets, were put into empty wine casks, and rolled into the Rhine, lest they should infect the air. The mob was forbidden to enter the ruins of the habitations that were burnt in the Jewish quarter; for the senate itself caused search to be made for the treasure, which is said to have been very considerable. At Strasburg, two thousand Jews were burnt alive in their own burial-ground, where a large scaffold had been erected: a few who promised to embrace Christianity were spared, and their children taken from the pile. The youth and beauty of several females also excited some commiseration; and they were snatched from death against their will: many, however, who forcibly made their escape from the flames were murdered in the streets." 110.

And such is uneducated man when his passions are let loose, excelling in ferocity the tiger, and in cunning the serpent. A very graphic description of the moral, or rather immoral effects of the pestilence in Italy is given by Boccacio. We regret that we have not space for it.

We fancy that we need not inform our readers that the causes of this, as of every other epidemic are involved in impenetrable mystery. The facts are—that it originated in Asia, that it spread from East to West, that it was preceded and accompanied by unusual and destructive terrestrial and aerial changes, and that it appeared to be contagious. Further than this all is an ocean of uncertainty, where the dove of science finds no resting place. The contagionist may hug himself in the idea that it was merely a contagious malady, the anti-contagionist may see all in the earth or in the air, but we fear that the great causes of these epidemic visitations are likely to remain as hidden as they have ever been, and baffle the application of reasoning drawn from ordinary facts. The Faculty of Medicine of Paris were called upon, as Faculties have been in later days, to display their collective wisdom. Whether the ancient or the modern corporations have acquitted themselves best, will be seen from the following amusing manifesto issued by the learned Thebans of the 14th century. It will be observed that the tone of the document is like that of more recent proclamations, somewhat dogmatic.

"We, the Members of the College of Physicians, of Paris, have after mature consideration and consultation on the present mortality, collected the advice of our old masters in the art, and intend to make known the causes of this pestilence more clearly than could be done according to the rules and principles of astrology and natural science; we, therefore, declare as follows:—

It is known that in India, and the vicinity of the Great Sea, the constellations which combated the rays of the sun, and the warmth of the heavenly fire, exerted their power especially against that sea, and struggled violently with its waters. Hence, vapours often originate which envelope the sun, and convert his light into darkness. These vapours alternately rose and fell for twenty-eight days; but at last sun and fire acted so powerfully upon the sea, that they attracted a great portion of it to themselves, and the waters of the ocean arose in the form of vapour; thereby the waters were in some parts so corrupted, that the fish which they contained died. These corrupted waters, however, the heat of the sun could not consume, neither could other wholesome water, hail or snow, and dew, originate therefrom. On the contrary, this vapour spread itself through the air in many places on the earth, and enveloped them in fog.

Such was the case all over Arabia, in a part of India; in Crete; in the plains and valleys of Macedonia; in Hungary; Albania and Sicily. Should the same thing occur in Sardinia, *not a man will be left alive;* and the like will continue, so long as the sun remains in the sign of Leo, on all the islands and adjoining countries to which this corrupted sea-wind extends, or has already extended from India. If the inhabitants of those parts do not employ and adhere to the following, or similar means and precepts, we announce to them inevitable death—except the grace of Christ preserve their lives.

We are of opinion, that the constellations, with the aid of Nature, strive, by virtue of their divine might, to protect and heal the human race; and to this end, in union with the rays of the sun, acting through the power of fire, endeavour to break through the mist. Accordingly, within the next ten days, and until the 17th of the ensuing month of July, this mist will be converted into a stinking deleterious rain, whereby the air will be much purified. Now, as soon as this rain announces itself, by thunder or hail, every one of you should protect himself from the air; and, as well before as after the rain, kindle a large fire of vine-wood, green laurel, or other green wood; worm-wood and chamomile should also be burnt in great quantity in the market places, in other densely inhabited localities, and in the houses. Until the earth is again completely dry, and for three days afterwards, no one ought to go abroad in the fields. During this time the diet should be simple, and people should be cautious in avoiding exposure in the cool of the evening, at night, and in the morning. Poultry and water-fowl, young pork, old beef, and fat meat, in general, should not be eaten; but, on the contrary, meat of a proper age, of a warm and dry nature, by no means, however, heating and exciting. Broth should be taken, seasoned with ground pepper, ginger and cloves, especially by those who are accustomed to live temperately, and are yet choice in their diet. Sleep in the day-time is detrimental; it should be taken at night until sun-rise, or somewhat longer. At breakfast one should drink little; supper should be taken an hour before sunset, when more may be drunk than in the morning. Clear light wine, mixed with a fifth or sixth part of water, should be used as a beverage. Dried or fresh fruits with wine are not injurious; but highly so without it. Beet-root and other vegetables, whether eaten pickled or fresh, are hurtful; on the contrary, spicy pot-berbs, as sage or rosemary, are wholesome. Cold, moist, watery food is, in general, prejudicial. Going out at night, and even until three o'clock in the morning, is dangerous, on account of the dew. Only small river fish should be used. Too much exercise is hurtful.. The body should be kept warmer than usual, and thus protected from moisture and cold. Rain-water must not be employed in cooking, and every one should guard against exposure to wet weather. If it rain, a little fine treacle should be taken after dinner. *Fat people should not sit in the sunshine.* Good clear wine should be selected and drunk often, but in small quantities, by day. Olive oil, as an article of food, is fatal. Equally injurious are fasting or excessive abstemiousness, anxiety of mind, anger, and excessive drinking. Young people, in Autumn especially, must abstain from all these things, if they do not wish to run a risk of dying of dysentery. In order to keep the body properly open, an enema, or some other simple means, should be employed, when necessary. Bathing is injurious. Men must preserve chastity as they value their lives. Every one should impress this on his recollection, but especially those who reside on the coast, or upon an island into which the noxious wind has penetrated.' "* 135.

* "*Jacob. Franciscliini de Ambrosiis.* In the Appendix to the Istorie Pistolesi. *Muratori*, Tom. XI. p. 528."

We recommend this document to the attentive consideration of those who may be called on for similar purposes. The easy manner in which general principles are blended with special recommendations is highly creditable to the Parisian sages. Our author gives a very interesting account of the origin of quarantine regulations, but for this we cannot afford space at present. We have given the principal points connected with this formidable plague, and, as the newspapers inform us that the influenza, under which we are now suffering, is to be the forerunner of some dire pestilence, we have enabled the alarmed or the alarmists to see what occurred in other times.

